# Metagenome Sequencing of the Microbial Community of a Solar Saltern Crystallizer Pond at Cáhuil Lagoon, Chile

**DOI:** 10.1128/genomeA.01172-14

**Published:** 2014-11-13

**Authors:** Alvaro M. Plominsky, Nathalie Delherbe, Juan A. Ugalde, Eric E. Allen, Marine Blanchet, Priscila Ikeda, Francisco Santibañez, Kurt Hanselmann, Osvaldo Ulloa, Rodrigo De la Iglesia, Peter von Dassow, Marcia Astorga, María Jesús Gálvez, María Lorena González, Carlos Henríquez-Castillo, Daniel Vaulot, Adriana Lopes do Santos, Gerrit van den Engh, Carla Gimpel, Florencia Bertoglio, Yolaine Delgado, Felipe Docmac, Claudia Elizondo-Patrone, Silvia Narváez, Fernando Sorroche, Marcelo Rojas-Herrera, Nicole Trefault

**Affiliations:** a Facultad de Ciencias Biológicas, Pontificia Universidad Católica de Chile, Santiago, Chile; b Facultad de Ciencias, Universidad Mayor, Santiago, Chile; c Marine Biology Research Division, Scripps Institution of Oceanography, University of California San Diego, San Diego, California, USA; d Division of Biological Sciences, University of California San Diego, San Diego, California, USA; e Biogeochemistry and Microbiology, Université Pierre et Marie Curie, Paris, France; f Oceanographic Institute, University of Sao Paulo, São Paulo, Brazil; g Departamento de Oceanografía, Universidad de Concepción, Concepción, Chile; h Department of Earth Sciences, Climate Geology, Geomicrobiology, ETH Zürich, Zürich, Switzerland; i Instituto Milenio de Oceanografía, Universidad de Concepción, Concepción, Chile; j Sorbonne Universités, UPMC Université Paris 06, UMR 7144, Station Biologique, Roscoff, France; k CNRS, UMR 7144, Station Biologique, Roscoff, France; l Federal University of Rio de Janeiro, Rio de Janeiro, Brazil; m BD Advanced Cytometry Group, Seattle, Washington, USA; n Departamento Científico, Instituto Antártico Chileno, Punta Arenas, Chile; o Centro Universitario Regional Este, Universidad de la República, Rocha, Uruguay; p Departamento de Microbiología, Universidad de La Habana, La Habana, Cuba; q Facultad de Ciencias del Mar y Recursos Biológicos, Universidad de Antofagasta, Antofagasta, Chile; r Departamento de Ecología y Recursos Naturales, Universidad Andrés Bello, Santiago, Chile; s Instituto de Investigaciones Marinas, INVEMAR, Santa Marta, Colombia; t Facultad de Ciencias Exactas, Físico-Químicas y Naturales, Universidad Nacional de Río Cuarto, Córdoba, Argentina

## Abstract

Cáhuil Lagoon in central Chile harbors distinct microbial communities in various solar salterns that are arranged as interconnected ponds with increasing salt concentrations. Here, we report the metagenome of the 3.0- to 0.2-µm fraction of the microbial community present in a crystallizer pond with 34% salinity.

## GENOME ANNOUNCEMENT

Solar salterns represent unique extreme microbial ecosystems that are best studied by applying different molecular techniques (1). These habitats offer relatively low microbial diversity and are generally dominated by archaea (2). In this study, we generated an additional data set from a solar saltern crystallizer pond in Cáhuil Lagoon (Chile). We will continue to use the data for comparative studies on the metabolic potential and diversity of microorganisms inhabiting an environment with 10-fold seawater salt concentration.

Cáhuil Lagoon extends several miles into mainland from the coast of the South Pacific, where seawater mixes with various freshwater tributaries. Along the border of the lagoon, local inhabitants build a series of locks to distribute brackish water into a series of interconnected ponds where water evaporates until the ion activity products of the dissolved ions exceed the solubility product of halite.

Samples for this study were collected on 12 January 2012 from a final salt crystallizer pond (34°30′10″S 71°59′09″W), which had a salinity of 34.1%, a temperature of 41.2°C, and a pH of 6.4. A volume of 5 liters was first passed through a 60-µm nylon mesh, and then 600 ml was sequentially filtered through 20-µm, 3.0-µm (polycarbonate; Millipore), and 0.22-µm-pore filters (polyethersulfone; Millipore). Community DNA was extracted from the 3.0- to 0.22-µm size fraction using the cetyltrimethylammonium bromide (CTAB) method (3) and sequenced by pyrosequencing (Roche 454 GS-FLX system, Titanium chemistry) at the Center for Genomics and Bioinformatics, Universidad Mayor, Chile.

The raw 222,074 sequencing reads (average length, 450 bp) were quality filtered for ambiguities and homopolymers (≥10), and reads with >75% of the sequence having a Phred quality score of ≤20 were removed. Taxonomic assignment (MGTAXA [http://mgtaxa.jcvi.org] against NCBI RefSeq v01-07-2012) of the remaining 183,299 reads revealed that 61% stem from archaea, 19% from viruses, and 16% from bacteria. At the phylum level, 58% of the reads were assigned to *Euryarchaeota* (all from the family *Halobacteriaceae*), 9% to *Actinobacteria*, and 7% to the virus class *Caudovirales*. Several genera of the family *Halobacteriaceae* share similar levels of representation (expressed as the % of reads assigned to *Archaea*): *Haloarcula* (14%), *Haloquadratum* (11.5%), *Halorubrum* (9.5%), *Halobacterium* (9%), *Natronomonas* (9%), *Halogeometricum* (8.5%), *Halomicrobium* (6.5%), and *Halorhabdus* (6%). Although *Haloquadratum* is still an important part of the microbial community, it is not as abundant in this crystallizer pond as it is in the Santa Pola saltern (1, 4).

The data generated as part of this metagenome add valuable information for ongoing and future studies on the metabolic potential of the microbial communities associated with extreme hypersaline habitats worldwide.

### Nucleotide sequence accession number.

This metagenome project has been deposited in the NCBI Sequence Read Archive under the accession no. SRX680116.
